# Evaluating AAPM‐TG‐218 recommendations: Gamma index tolerance and action limits in IMRT and VMAT quality assurance using SunCHECK

**DOI:** 10.1002/acm2.14277

**Published:** 2024-01-19

**Authors:** Jia Deng, ShengYan Liu, Yun Huang, Xiuquan Li, Xiangyang Wu

**Affiliations:** ^1^ Department of Radiation Oncology Shaanxi Provincial Tumor Hospital Xian Shaanxi China; ^2^ School of Nuclear Science and Technology Xi'an Jiaotong University Xi'an Shaanxi China; ^3^ Department of Radiation Oncology Yulin Xingyuan Hospital Xi'an China; ^4^ Department of Oncology Second Affiliated Hospital of Guizhou University of Traditional Chinese Medicine Guiyang City Guizhou Province China; ^5^ Department of Oncology The First Affiliated Hospital of Chongqing Medical University Chongqing China

**Keywords:** action limits, gamma passing rate, IMRT QA, VMAT QA

## Abstract

**Purpose:**

This study aimed to improve the safety and accuracy of radiotherapy by establishing tolerance (TL) and action (AL) limits for the gamma index in patient‐specific quality assurance (PSQA) for intensity‐modulated radiation therapy (IMRT) and volumetric‐modulated arc therapy (VMAT) using SunCHECK software, as per AAPM TG‐218 report recommendations.

**Methods:**

The study included 125 patients divided into six groups by treatment regions (H&N, thoracic and pelvic) and techniques (VMAT, IMRT). SunCHECK was used to calculate the gamma passing rate (%GP) and dose error (%DE) for each patient, for the planning target volume and organs at risk (OARs). The TL and AL were then determined for each group according to TG‐218 recommendations. We conducted a comprehensive analysis to compare %DE among different groups and examined the relationship between %GP and %DE.

**Results:**

The TL and AL of all groups were more stringent than the common standard as defined by the TG218 report. The TL and AL values of the groups differed significantly, and the values for the thoracic groups were lower for both VMAT and IMRT. The %DE of the parameters D_95%_, D_90%_, and D_mean_ in the planning target volume, and D_mean_ and D_max_ in OARs were significantly different. The dose deviation of VMAT was larger than IMRT, especially in the thoracic group. A %GP and %DE correlation analysis showed a strong correlation for the planning target volume, but a weak correlation for the OARs. Additionally, a significant correlation existed between %GP of SunCHECK and Delta4.

**Conclusion:**

The study established TL and AL values tailored to various anatomical regions and treatment techniques at our institution. Establishing PSQA workflows for VMAT and IMRT offers valuable clinical insights and guidance. We also suggest developing a standard combining clinically relevant metrics with %GP to evaluate PSQA results comprehensively.

## 1 INTRODUCTION

The implementation of patient‐specific quality assurance (PSQA) is important in the execution of intensity‐modulated radiation therapy (IMRT) and volumetric‐modulated arc therapy (VMAT).^1^, ^2^, ^3^ PSQA plays a crucial role in evaluating the congruence between intended and actual administered doses. In addition, it can detect discrepancies during the treatment planning process, mitigate potential errors in both IMRT and VMAT, guarantee patient safety and wellbeing during the treatment process, and ensure successful execution of the treatment plan.^4^, ^5^

The radiotherapy planning process is subject to uncertainty owing to a multitude of circumstances. From the perspective of treatment planning, uncertainties may arise from the leaf end effect, tongue‐and‐groove effect resulting from the unique form of the multi‐leaf collimator, collimator transmission factor, penumbra, field output factor, and dosimetry grid size.^6^, ^7^, ^8^

With radiation therapy delivery, uncertainty arises from factors such as errors in multi‐leaf collimator leaf positioning, instability in gantry rotation, instability of the treatment couch, fluctuations in beam stability, and accelerator utilization time.^9^, ^10^

Following dose verification, it is feasible to conduct a comparative analysis of outcomes by overlaying isodose line distributions; however, visual quantification of the disparity is impractical. Hence, as indicated in the American Association of Physicists in Medicine Task Group (AAPM‐TG) 218 report, the recommended approach for assessing dose verification outcomes is the gamma passing rate (%GP) technique.^11^ %GP is determined by evaluating the dose and distance deviations.^12^ TG‐218 suggests adopting a more stringent criterion of 3%/2 mm, as opposed to the 3%/3 mm norm proposed in the TG‐119 report.^13^

TG‐218 recommends the adoption of tolerance (TL)‐ and action limit (AL)‐based workflows for the comprehensive monitoring of the status of all IMRT/VMAT QA specifications. Furthermore, it suggests standardizing the TLs and ALs to enhance consistency and reliability. Considering the complexity of radiotherapy planning for different anatomical sites in the clinic, it is necessary to establish customized tolerance and intervention limits for each specific site.^11^

The report also suggested that the clinical significance of the dose error (%DE) should be assessed if %GP is below a specified threshold.^11^ However, most PSQA devices currently used in treatment facilities do not yield clinically relevant outcomes.^14^ Further, most of these devices are based on phantoms, rather than on actual patient CT images. In contrast, the SunCHECK system can deliver pertinent results by leveraging patient CT scans and treatment records from an accelerator.^15^

This study implemented AAPM TG‐218 recommendations using the SunCHECK system to set TL and AL for %GP in PSQA for the head and neck (HNC), thoracic, and pelvis, as well as VMAT and IMRT techniques. This study also assessed the clinical significance of %GP, thereby enhancing the accuracy of radiation therapy techniques. Additionally, Delta4 was used to perform PSQA on the same patients to verify the reliability of the SunCHECK software results.

## 2 MATERIALS AND METHODS

### 2.1 Patient selection

A total of 125 patients were analyzed in the study. These included 36 cases of HNC, including nasopharyngeal cancer, whole‐brain and neck tumors, with 20 cases treated using VMAT and 16 cases with IMRT; 40 cases of thoracic cancer (TC), including esophageal and lung cancers, with 20 cases treated using VMAT and 20 cases with IMRT; and 49 cases of pelvis cancer (PC), including cervical and bladder cancers, with 37 cases treated using VMAT and 12 cases treated using IMRT. As shown in Table 1, the cases were categorized into six groups. An anisotropic analytical algorithm (AAA) within the Varian Eclipse treatment planning system (TPS) was used for dose calculation, with a computational grid of 2.5 mm and beam energy of 6 MV. All the plans were executed using a Varian TrueBeam accelerator.

**TABLE 1:** Case grouping and plan information.

Group	HNC‐VMAT	HNC‐IMRT	TC‐VMAT	TC‐IMRT	PC‐VMAT	PC‐IMRT
Energy	6 MV	6 MV	6 MV	6 MV	6 MV	6 MV
No. of plans	20	16	20	20	37	12
Prescribed dose (Gy)	5940–7000	3000–5700	5000	5000–6000	4600–5000	4600–6000

Abbreviations: HNC, head and neck cancer; IMRT, intensity‐modulated radiation therapy; PC, pelvis cancer; TC, thoracic cancer; VMAT, volumetric‐modulated arc therapy.

### 2.2 SunCHECK system patient quality assurance

SunCHECK is an automated QA system made by Sun Nuclear. Within SunCHECK, the Patient module is responsible for patient quality management using accelerator log files, patient CT scans, and plan files, all powered by the Sun Nuclear dose calculator (SDC) algorithm.^16^

The process involves transferring plan files, such as RTPLAN, CT, RTSTRUCTURE, and RTDOSE, to SunCHECK which automatically generates the patient profile for planning and independent dose verification. While the cases are being implemented on the accelerator, SunCHECK automatically captures log files for the entire treatment process. It selects a pre‐defined verification template from the system, which primarily encompasses various pass rate criteria (Supplementary Appendix 1). Subsequently, it calculates and provides the global 3D %GP, 3D dose distribution, and dose‐volume histogram (DVH).^15^ SunCHECK's dose calculation is driven by the SDC, utilizing a Superposition/Convolution(CS) style. The method encompasses fluence calculation within the accelerator head, TERMA calculation from the accelerator to the patient, and a concluding superposition step for radiation transport inside the patient.^17^, ^18^

### 2.3 Calculation of TL and AL

TL and AL were computed according to the guidelines outlined in TG‐218^11^. Twenty treatment plans were randomly chosen and subjected to PSQA evaluation. These plans exhibited diverse levels of complexity, and all plans underwent duplicate measurements to mitigate potential uncertainties stemming from distinct conditions. This approach aimed to confirm the absence of conspicuous irregularities during the assessment process. The calculations for TL and AL were performed as follows^11^:

(1) $$TL\;=\bar{x}\;-2.660\cdot \frac{1}{n-1}\sum_{i\;=\;2}^{n}\left|x_{i}-x_{i-1}\right|,$$

(2) $$AL\;=\;100\%-\beta \sqrt{\sigma^{2}+{\left(\bar{x}-T\right)}^{2}}/2,$$

where $\bar{x}$ is the average of %GP, *n* represents the number of plans, $\sigma^{2}$ represents the variance, *T* represents the ideal value (set at 100), and β is a constant with a value of 6.0^19^.Subsequently, statistical process control charts and various evaluation criteria were established using the TL, the AL, the mean value (center line, CL), and %GP as parameters.

### 2.4 Delta4

The PSQA was performed using the Delta4 system, a three‐dimensional dose verification device developed by ScandiDos, to verify the reliability of the SunCHECK results. It incorporates a dual orthogonal semiconductor detector array in a cylindrical Polymethyl Methacrylate (PMMA) mold 22 cm in diameter and 40 cm in length. The system has 1069 detector points spaced 5 mm apart at the center and 10 mm apart at the periphery.^5^ For each case, clinical plans were applied to the Delta4 phantom, and then the recalculated plans and dose distribution files were transferred to the Delta4 system. After measurement, the %GP was calculated and recorded for evaluation.^20^

### 2.5 Data collection and analysis

The Suncheck system was used to collect the PSQA results from 125 cases. The mean dose (D_mean_), the maximum dose (D_max_), and dose to 95% (D_95%_) of the planned target volume (PTV) were calculated and compared.

Dose distribution statistics were computed for the parameters outlined in Table 2 and compared with the dose distribution in the TPS to calculate the %DE using the following formula:

(3) $$\%DE\;=\;\left|\frac{D_{SunCHECK}-D_{TPS}}{D_{TPS}}\right|\times 100\%,$$

where $D_{SunCHECK}$ represents the dose distribution value reconstructed using SunCHECK and $D_{TPS}$ represents the dose value obtained from the planning system.

**TABLE 2:** DVH parameters in different anatomical structure groups.

	Structure	DVH parameter		
HNC	PTV	D_95%_	D_90%_	D_mean_
	Spinal cord	D_mean_	D_max_	
	Brian stem	D_mean_	D_max_	
TC	PTV	D_95%_	D_90%_	D_mean_
	Heart	D_mean_	D_max_	
	Lungs	D_mean_	D_max_	
PC	PTV	D_95%_	D_90%_	D_mean_
	Rectum	D_mean_	D_max_	
	Bladder	D_mean_	D_max_	

Abbreviations: DVH, dose‐volume histogram; HNC, head and neck cancer; PC, pelvis cancer; PTV, planned target volume; TC, thoracic cancer.

The 3D %GP in SunCHECK and Delta4 were assessed using the standard 3%/3 mm criteria from TG119^13^, the standard 3%/2 mm criteria from TG‐218, and the more stringent criteria of 2%/2  and 1%/1 mm^11^.

Linear regression and Pearson's correlation analyses were used to examine the correlation between %DE and %GP. The results from SunCHECK and Delta4 were subjected to both linear regression and Pearson's correlation analyses for further evaluation.

## 3 RESULTS

### 3.1 SunCHECK %GP

The average %GP for the six groups calculated in SunCHECK, using the four different threshold criteria, is listed in Table 3. As the pass criteria becomes tighter, the %GP values exhibited a declining trend. The average %GP of the PC group was higher than that of the HNC and TC groups, whereas the average %GP for VMAT in the HNC and TC groups was lower than that in the IMRT group.

**TABLE 3:** Mean %GP and variation for different criteria.

	%$\bar{GP}$±SD%			
Group	3%3 mm	3%2 mm	2%2 mm	1%1 mm
HNC‐VMAT	99.17 ± 0.82	96.91 ± 1.36	94.36 ± 2.09	74.19 ± 11.39
HNC‐IMRT	99.41 ± 0.41	98.41 ± 0.94	95.10 ± 3.30	78.53 ± 9.14
TC‐VMAT	97.67 ± 1.59	95.06 ± 2.63	92.56 ± 3.48	77.03 ± 5.08
TC‐IMRT	99.09 ± 0.76	97.87 ± 1.54	95.91 ± 2.60	80.65 ± 5.26
PC‐VMAT	99.53 ± 0.38	99.16 ± 0.45	98.13 ± 0.64	86.88 ± 2.56
PC‐IMRT	99.49 ± 0.84	98.92 ± 1.12	97.08 ± 2.22	79.59 ± 7.87

Abbreviations: %GP, gamma passing rate; HNC, head and neck cancer; IMRT, intensity‐modulated radiation therapy; PC, pelvis cancer; TC, thoracic cancer; VMAT, volumetric‐modulated arc therapy.

**FIGURE 1:**
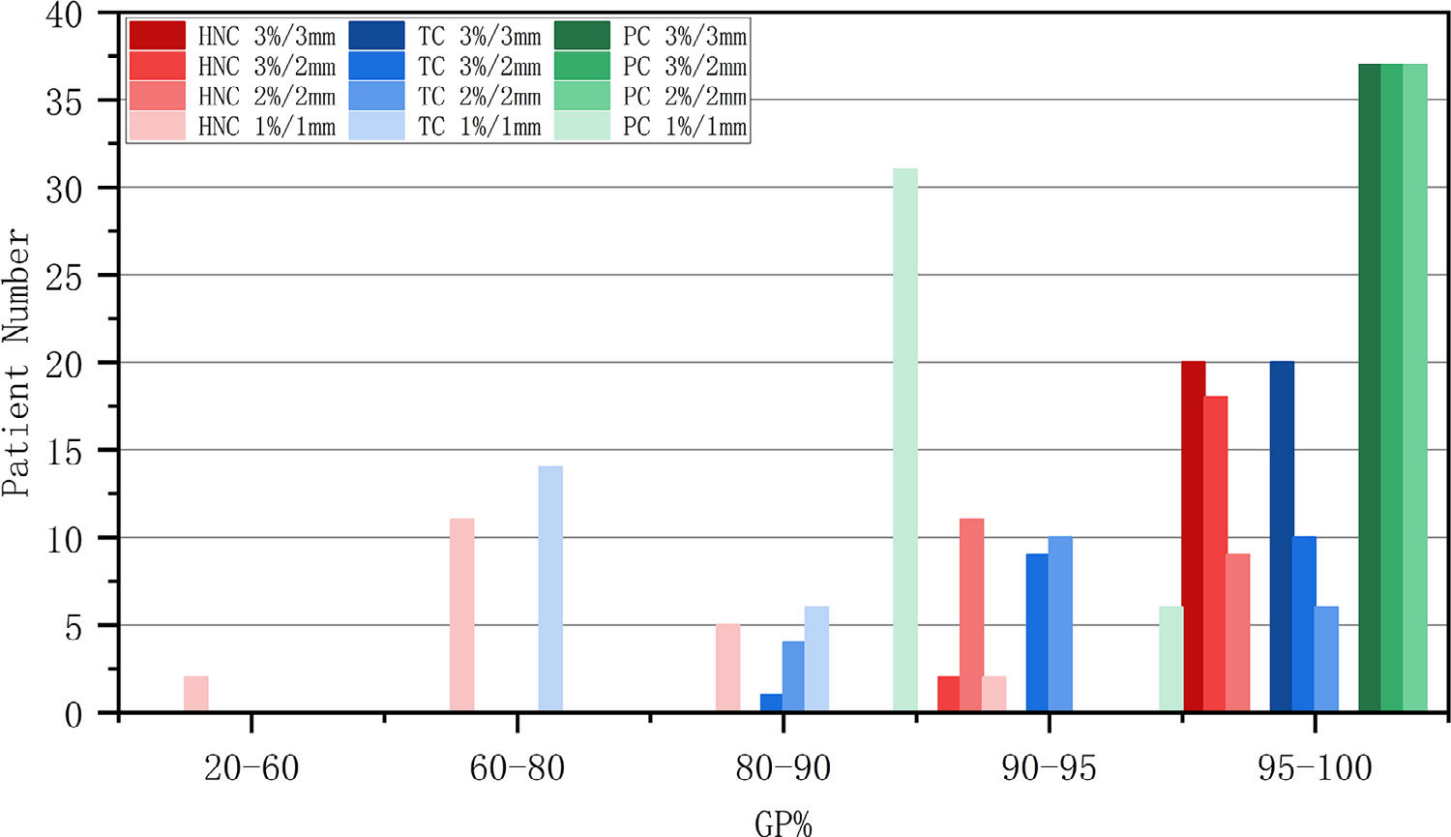
Distribution of %GP using VMAT for different anatomical structure groups. %GP, gamma passing rate; VMAT, volumetric‐modulated arc therapy.

**FIGURE 2:**
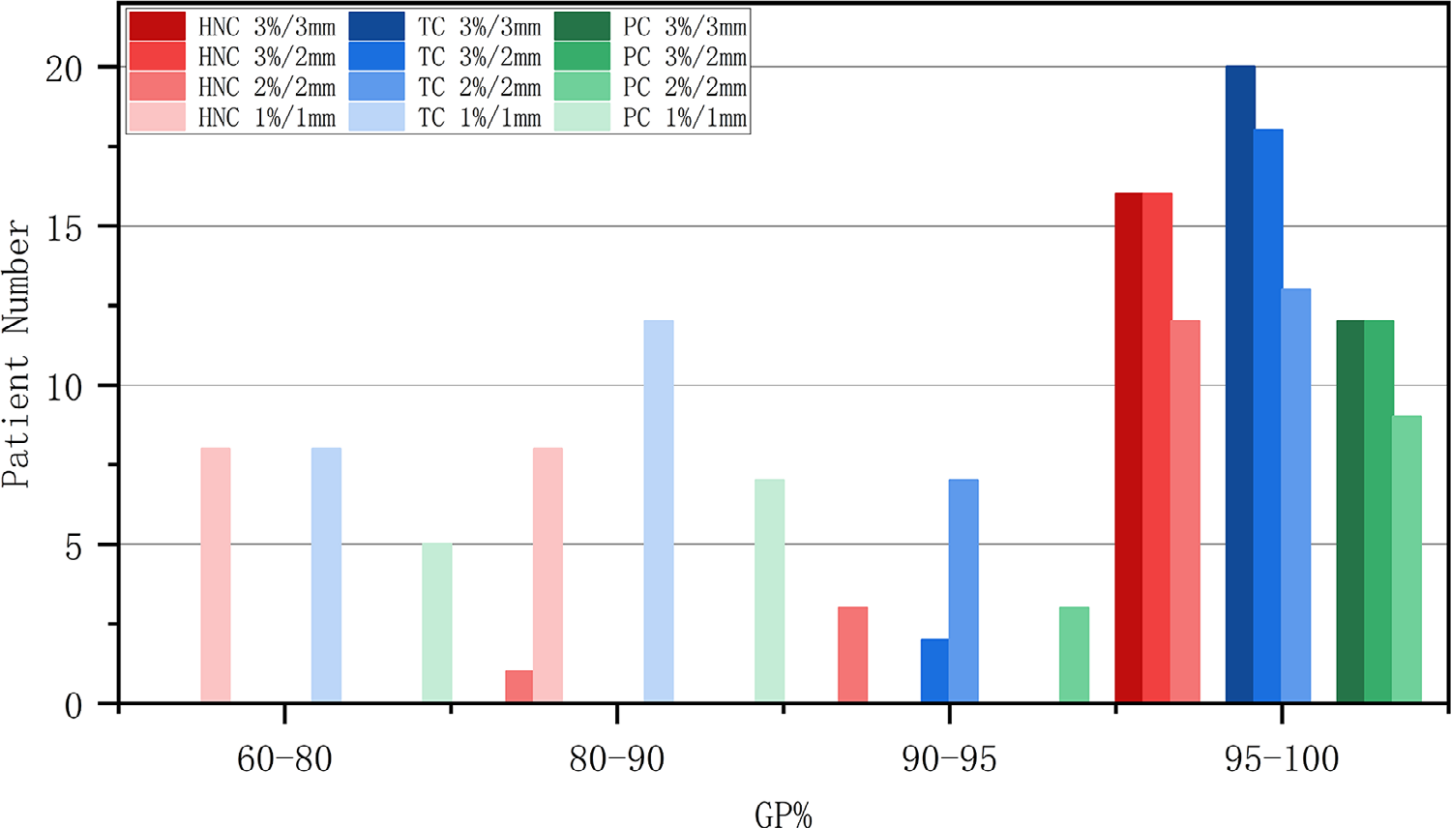
Distribution of %GP using IMRT for different anatomical structure groups. %GP, gamma passing rate; IMRT, intensity‐modulated radiation therapy.

The %GP distribution in different groups with different threshold criteria is shown in Figures 1 and 2. The HNC and TC groups included a higher proportion of patients with low %GP values than the PC group.

### 3.2 Statistical process control analysis

**FIGURE 3:**
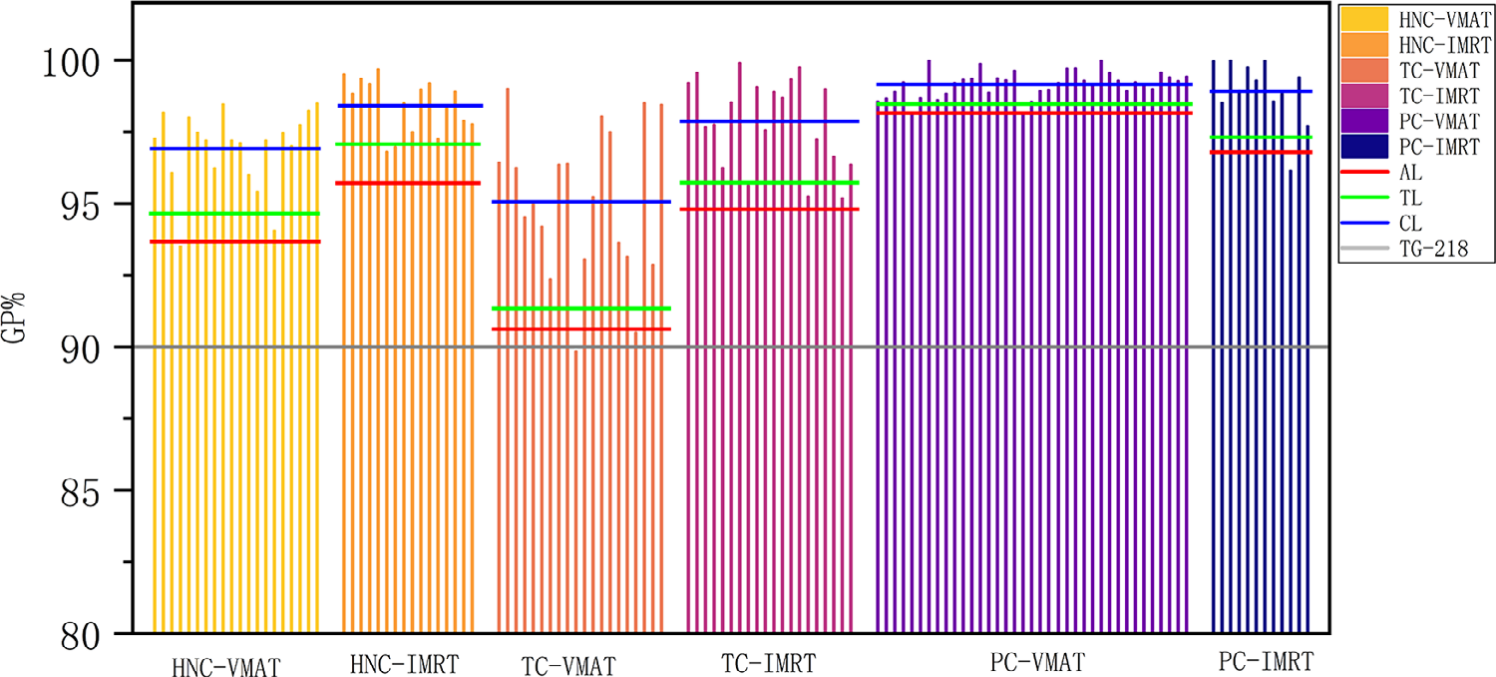
PSQA statistical process control chart for VMAT and IMRT groups across different anatomical sites under the 3%/2 mm criterion: representing AL, TL, CL, and the TG‐218 AL of 90%. AL, action limit; CL, center line; IMRT, intensity‐modulated radiation therapy; PSQA, patient‐specific quality assurance; TL, tolerance limit; VMAT, volumetric‐modulated arc therapy.

Twenty patients from each group were randomly selected for PSQA and their CLs, TLs, and Als were calculated. Table 4 presents the distribution range of gamma pass rates under the 3%2 mm acceptance criteria, along with the TL, AL, and CL. Figure 3 shows the Statistical Process Control Chart results of the PSQA for VMAT and IMRT groups across three different sites, under the 3%2 mm criteria.^19^, ^21^ Most of these values exceeded the standard reported by TG‐218. Both the HNC‐IMRT and TC‐IMRT groups demonstrated higher passing rates than their VMAT counterparts, except for one case in the TC‐VMAT group, which failed to meet the AL standard. However, the %GP of PC‐VMAT was higher than that of PC‐IMRT, and one case of PC‐IMRT group did not meet the AL standard.

**TABLE 4:** Distribution range of %GP, TL, AL, and CL under 3%2 mm criterion.

Group	Range (%)	TL (%)	AL (%)	CL (%)
HNC‐VMAT	93.50–98.49	94.65	93.68	96.91
HNC‐IMRT	96.8–99.68	97.07	95.71	98.41
TC‐VMAT	89.85–99	91.34	90.62	95.06
TC‐IMRT	95.19–99.90	95.72	94.80	97.87
PC‐VMAT	98.12–99.98	98.46	98.16	99.16
PC‐IMRT	96.14–99.99	97.31	96.79	98.92

Abbreviations: AL, action limit; CL, center line; %GP, gamma passing rate; HNC, head and neck cancer; IMRT, intensity‐modulated radiation therapy; PC, pelvis cancer; TL, tolerance limit, VMAT, volumetric‐modulated arc therapy.

### 3.3 Relative dosimetric error

**FIGURE 4:**
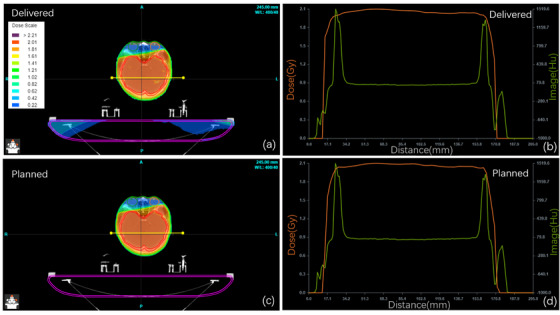
PSQA comparison for a whole brain VMAT case: SunCHECK dose distributions (a, b) versus planned doses (c, d), with cross‐sections in (a, c) (yellow line) corresponding to dose profiles in (b, d) (orange line) and HU distributions (green line). PSQA, patient‐specific quality assurance; VMAT, volumetric‐modulated arc therapy.

**FIGURE 5:**
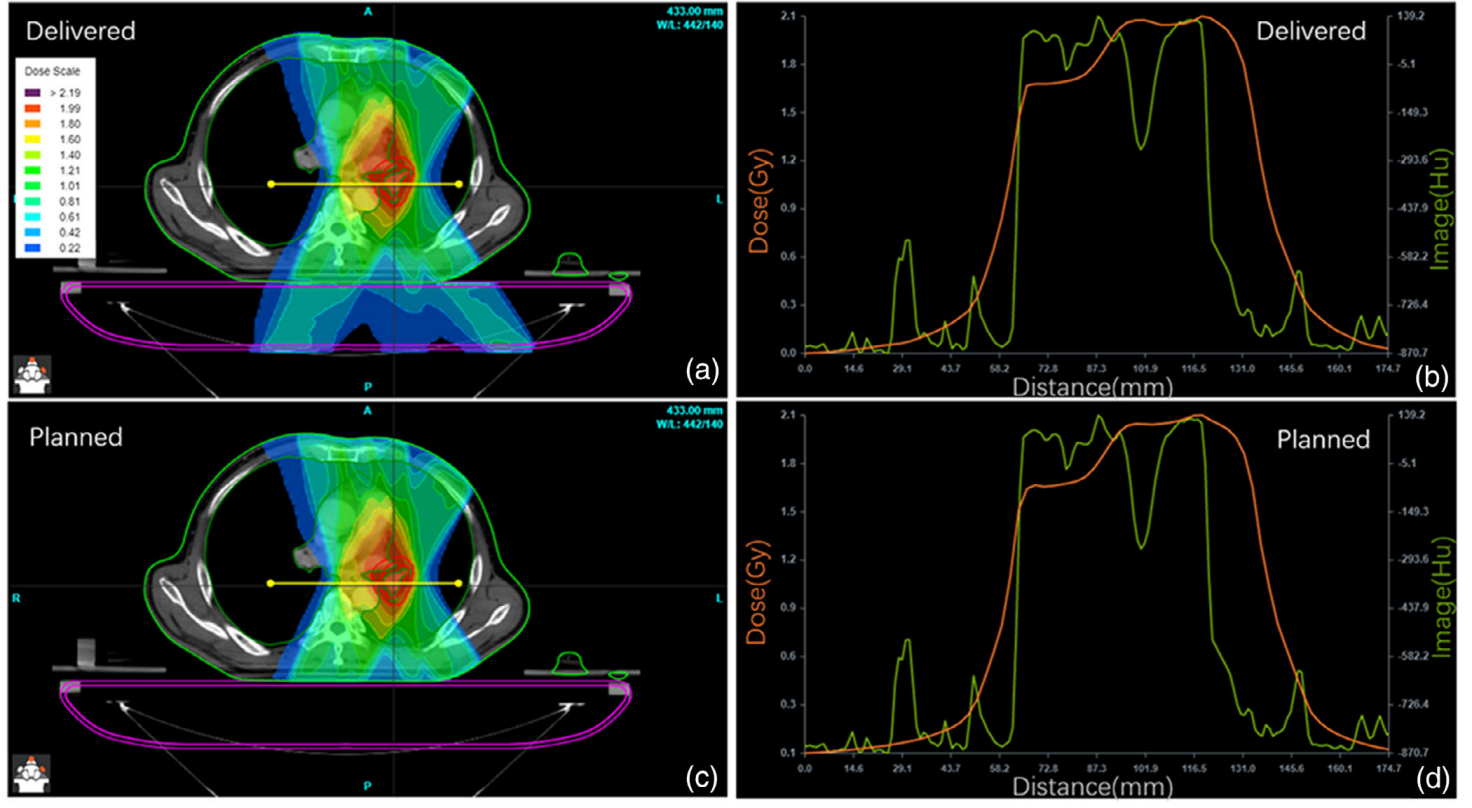
PSQA comparison for a lung cancer IMRT case: SunCHECK dose distributions (a, b) versus planned doses (c, d), with cross‐sections in (a, c) (yellow line) corresponding to dose profiles in (b, d) (orange line) and HU distributions (green line). IMRT, intensity‐modulated radiation therapy; PSQA, patient‐specific quality assurance.

**FIGURE 6:**
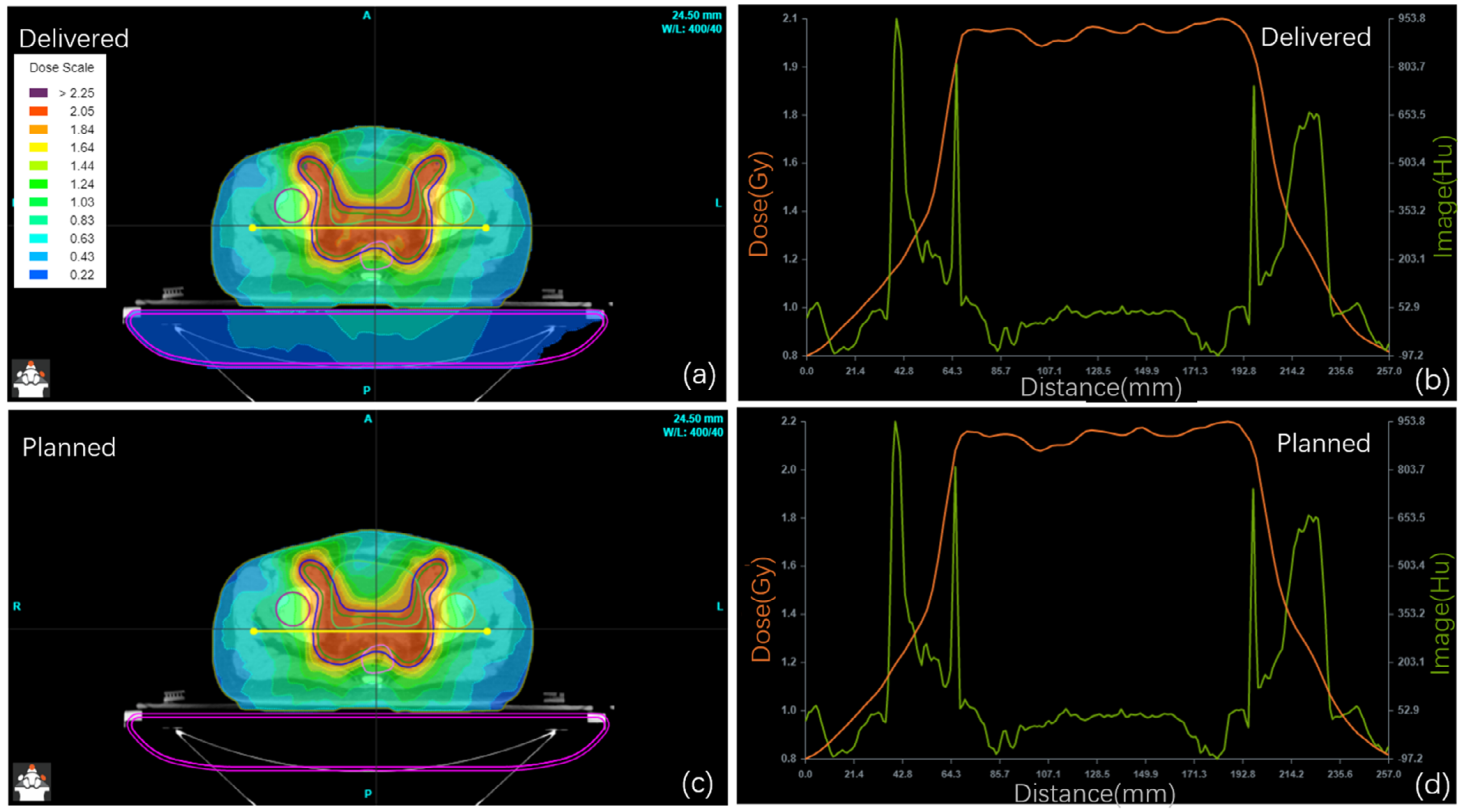
PSQA comparison for a cervical cancer VMAT case: SunCHECK dose distributions (a, b) versus planned doses (c, d), with cross‐sections in (a, c) (yellow line) corresponding to dose profiles in (b, d) (orange line) and HU distributions (green line). PSQA, patient‐specific quality assurance; VMAT, volumetric‐modulated arc therapy.

Figures 4, 5, and 6 show dose distribution comparisons between the dose verification and original plans for typical cases in the HNC, TC, and PC case groups, respectively. The doses were calculated using the log files and the SDC algorithm in SunCHECK and compared with the planned dose in the TPS. There were slight differences between SunCHECK and TPS, but the overall trends remained highly similar. In SunCHECK, a three‐dimensional dose distribution map was generated to facilitate the selection of regions of interest for dose comparison.

**FIGURE 7:**
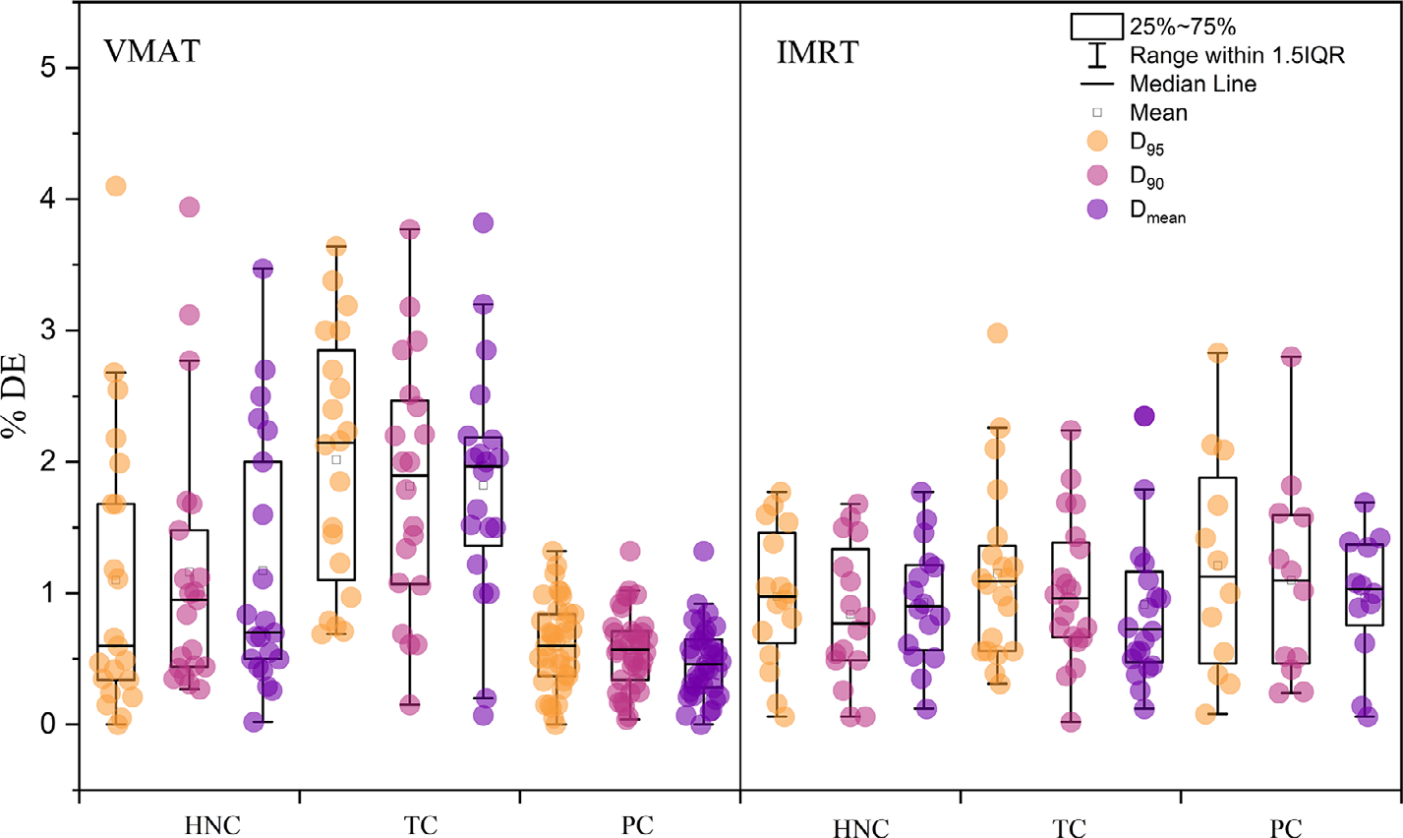
Boxplot comparison of PTV D_95%_, D_90%_, and D_mean_ dose deviations across three anatomical sites, contrasting VMAT and IMRT techniques with treatment planning and SunCHECK. IMRT, intensity‐modulated radiation therapy; PTV, planned target volume; VMAT, volumetric‐modulated arc therapy.

The mean and standard deviation of the %DE for D_95%_, D_90%_, and D_mean_ within the PTV across different groups were analyzed. The results are presented in Table 5 and their distribution is illustrated in Figure 7, which shows that different treatment techniques led to dose deviations. The %DE of VMAT was higher than that of IMRT in both the HNC and TC groups. In contrast, the %DE of VMAT was lower than that of IMRT in the PC group.

**TABLE 5:** PTV %DE between the TPS and SunCHECK.

	$\%\bar{DE}\pm SD\%\;$		
	PTV D_95%_	PTV D_90%_	PTV D_mean_
HNC‐VMAT	1.16 ± 1.09	1.17 ± 1.03	1.13 ± 0.98
HNC‐IMRT	0.98 ± 0.52	0.84 ± 0.53	0.93 ± 0.45
TC‐VMAT	2.02 ± 0.96	1.82 ± 0.97	1.82 ± 0.91
TC‐IMRT	1.15 ± 0.69	1.02 ± 0.55	0.91 ± 0.63
PC‐VMAT	0.60 ± 0.34	0.57 ± 0.30	0.48 ± 0.28
PC‐IMRT	1.21 ± 0.85	1.10 ± 0.77	0.97 ± 0.50

Abbreviations: HNC, head and neck cancer; IMRT, intensity‐modulated radiation therapy; PC, pelvis cancer; TPS, treatment planning system; VMAT, volumetric‐modulated arc therapy; PTV, planned target volume ; %DE, dose error; TC, thoracic cancer.

**FIGURE 8:**
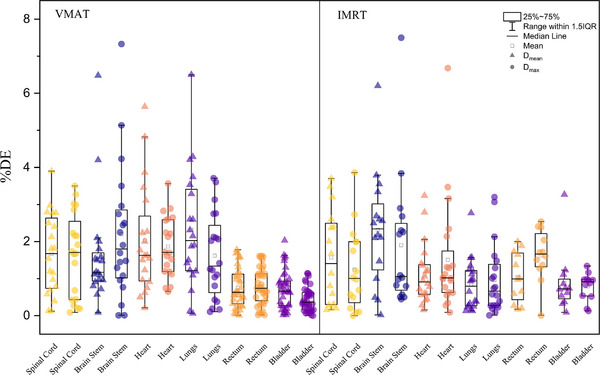
Distributions of organ‐at‐risk %DE for the TPS and SunCHECK. %DE, dose error; TPS, treatment planning system.

A comparison of the %DE of the OARs between the groups is shown in Table 6 and Figure 8. In general, %DE was higher in the VMAT group than in the IMRT group, especially in the TC group.

**TABLE 6:** Comparison of OAR %DE between the TPS and SunCHECK.

	$\%\bar{DE}\pm SD\%$			
	Spinal cord D_mean_	Spinal cord D_max_	Brain stem D_mean_	Brain stem D_max_
HNC‐VMAT	1.67 ± 1.08	1.65 ± 1.14	1.61 ± 1.41	2.20 ± 1.81
HNC‐IMRT	1.57 ± 1.27	1.24 ± 1.10	2.31 ± 1.53	1.90 ± 1.82

	$\%\bar{DE}\pm SD\%$			
	Heart D_mean_	Heart D_max_	Lungs D_mean_	Lungs D_max_
TC‐VMAT	2.03 ± 1.46	1.86 ± 0.82	2.31 ± 1.59	1.62 ± 1.15
TC‐IMRT	1.12 ± 0.81	1.51 ± 1.52	0.83 ± 0.67	0.96 ± 0.93

	$\%\bar{DE}\pm SD\%$			
	Rectum D_mean_	Rectum D_max_	Bladder D_mean_	Bladder D_max_
PC‐VMAT	0.76 ± 0.54	0.79 ± 0.49	0.71 ± 0.52	0.45 ± 0.32
PC‐IMRT	1.04 ± 0.67	1.63 ± 0.71	0.88 ± 0.82	0.79 ± 0.37

Abbreviations: %DE, dose error; HNC, head and neck cancer; IMRT, intensity‐modulated radiation therapy; OARs, organs at risk; PC, pelvis cancer; TC, thoracic cancer; TPS, treatment planning system; VMAT, volumetric‐modulated arc therapy.

### 3.4 Correlation analysis of %GP rates and %DE

The correlation coefficients *r* and *p* for PTV D_95%_, PTV D_90%_, PTV D_mean_, brainstem D_mean_, brainstem D_max_, spinal cord D_mean_, and spinal cord D_max_ in the HNC groups under different thresholds (3%/3 , 3%/2 , 2%/2 , 1%/1 mm) are shown in Table 7. PTV D_95%_, PTV D_90%_, and PTV D_mean_ exhibited statistically significant negative correlations (*r*: −0.67 ∼ −0.51, *p* < 0.05). BrainStem D_mean_ and BrainStem D_max_ demonstrated a strong negative correlation in most cases, which was also statistically significant (*r*: −0.6 ∼ −0.35, *p* < 0.05). There was a weak and non‐significant correlation between the D_mean_ and D_max_ of the Spinal Cord (*r* value close to zero).

**TABLE 7:** Correlation between %GP and %DE in the HNC groups.

		3%3 mm		3%2 mm		2%2 mm		1%1 mm	
HNC		VMAT	IMRT	VMAT	IMRT	VMAT	IMRT	VMAT	IMRT
PTV D_95%_	*r*	−0.67	−0.43	−0.67	−0.47	−0.69	−0.56	−0.61	−0.6
	*p*	<0.01	0.17	<0.01	0.12	<0.01	0.06	<0.01	0.04
PTV D_90%_	*r*	−0.69	−0.4	−0.67	−0.47	−0.73	−0.49	−0.61	−0.54
	*p*	<0.01	0.19	<0.01	0.13	<0.01	0.1	<0.01	0.07
PTV D_mean_	*r*	−0.51	−0.54	−0.67	−0.48	−0.66	−0.55	−0.67	−0.46
	*p*	<0.01	0.07	<0.01	0.12	<0.01	0.07	<0.01	0.13
Brainstem D_mean_	*r*	−0.58	0.36	−0.58	0.22	−0.6	0.05	−0.41	−0.37
	*p*	<0.01	0.25	<0.01	0.49	<0.01	0.87	<0.01	0.23
Brainstem D_max_	*r*	−0.35	0.34	−0.49	0.12	−0.67	0.64	−0.37	0.59
	*p*	0.04	0.27	<0.01	0.71	<0.01	0.02	0.02	0.04
Spinal cord D_mean_	*r*	0.2	−0.52	0.25	−0.58	0.25	−0.61	−0.02	−0.38
	*p*	0.24	0.09	0.13	0.05	0.13	0.04	0.93	0.23
Spinal cord D_max_	*r*	0.12	0.03	0.06	−0.07	0.07	0.26	−0.07	0.61
	*p*	0.49	0.93	0.72	0.82	0.66	0.41	0.67	0.03

Abbreviations: %DE, dose error; %GP, gamma passing rate; HNC, head and neck cancer; IMRT, intensity‐modulated radiation therapy; VMAT, volumetric‐modulated arc therapy.

The results for the TC groups are presented in Table 8. PTV D_95%_, PTV D_90%_, and PTV D_mean_ showed a strong negative correlation, and the correlation was statistically significant (*r*: −0.56 ∼ −0.83, *p* < 0.01). The correlation between Heart D_mean_ and Heart D_max_ was weak and insignificant (*r* close to zero). Lung D_mean_ and Lung D_max_ showed a negative correlation (*r*: −0.73 ∼ −0.53), especially at 2%2  and 1%1 mm, the correlation was significant.

**TABLE 8:** Correlation between %GP and %DE in the chest cancer groups.

		3%3 mm		3%2 mm		2%2 mm		1%1 mm	
TC		VMAT	IMRT	VMAT	IMRT	VMAT	IMRT	VMAT	IMRT
PTV D_95%_	*r*	−0.6	−0.77	−0.83	−0.72	−0.56	−0.77	−0.56	−0.76
	*p*	<0.01	<0.01	<0.01	<0.01	<0.01	<0.01	<0.01	<0.01
PTV D_90%_	*r*	−0.7	−0.8	−0.87	−0.73	−0.56	−0.72	−0.49	−0.76
	*p*	<0.01	<0.01	<0.01	<0.01	<0.01	<0.01	0.03	<0.01
PTV D_mean_	*r*	−0.71	−0.77	−0.75	−0.73	−0.55	−0.79	−0.52	−0.72
	*p*	<0.01	<0.01	<0.01	<0.01	<0.01	<0.01	0.02	<0.01
Heart D_mean_	*r*	−0.27	0.08	−0.28	0.13	−0.49	0.17	−0.18	−0.09
	*p*	0.25	0.77	0.22	0.63	0.03	0.54	0.46	0.75
Heart D_max_	*r*	−0.24	−0.19	−0.33	−0.23	−0.36	−0.11	−0.32	−0.38
	*p*	0.3	0.48	0.16	0.39	0.12	0.68	0.17	0.14
Lung D_mean_	*r*	−0.53	−0.3	−0.72	−0.13	−0.59	−0.18	−0.21	−0.17
	*p*	0.02	0.26	<0.01	0.64	<0.01	0.5	0.37	0.53
Lung D_max_	*r*	−0.73	−0.49	−0.69	−0.46	−0.66	−0.56	−0.71	−0.53
	*p*	<0.01	0.05	<0.01	0.07	<0.01	0.02	<0.01	0.04

Abbreviations: %DE, dose error; %GP, gamma passing rate; IMRT, intensity‐modulated radiation therapy; VMAT, volumetric‐modulated arc therapy.

The correlations in the PC group are presented in Table 9. PTV D_95%_, PTV D_90%_, and PTV D_mean_ showed a strong negative correlation (*r*: −0.7 ∼−0.9, *p* < 0.01). The correlation between the Rectum D_mean_ and Rectum D_max_ was weak and not significant.

**TABLE 9:** Correlation between %GP and %DE in the abdominal cancer groups.

		3%3 mm		3%2 mm		2%2 mm		1%1 mm	
PC		VMAT	IMRT	VMAT	IMRT	VMAT	IMRT	VMAT	IMRT
PTV D_95%_	*r*	−0.56	−0.68	−0.59	−0.69	−0.7	−0.84	−0.77	−0.83
	*p*	<0.01	<0.01	<0.01	<0.01	<0.01	<0.01	<0.01	<0.01
PTV D_90%_	*r*	−0.56	−0.8	−0.61	−0.79	−0.81	−0.83	−0.79	−0.82
	*p*	<0.01	<0.01	<0.01	<0.01	<0.01	<0.01	<0.01	<0.01
PTV D_mean_	*r*	−0.8	−0.82	−0.79	−0.86	−0.78	−0.9	−0.75	−0.79
	*p*	<0.01	<0.01	<0.01	<0.01	<0.01	<0.01	<0.01	<0.01
Rectum D_mean_	*r*	−0.49	−0.07	−0.51	−0.02	−0.48	0.05	−0.6	−0.05
	*p*	0.03	0.78	0.02	0.95	0.03	0.84	<0.01	0.85
Rectum D_max_	*r*	−0.33	−0.32	−0.42	−0.31	−0.01	−0.39	−0.06	−0.3
	*p*	0.16	0.18	0.07	0.19	0.98	0.09	0.8	0.21
Bladder D_mean_	*r*	−0.36	−0.08	−0.05	−0.03	−0.05	0.17	0.36	−0.02
	*p*	0.11	0.73	0.84	0.91	0.82	0.48	0.12	0.94
Bladder D_max_	*r*	0.29	0.49	−0.16	0.24	0.02	0.25	−0.21	0.26
	*p*	0.22	0.03	0.49	0.31	0.93	0.28	0.39	0.26

Abbreviations: %DE, dose error; %GP, gamma passing rate; IMRT, intensity‐modulated radiation therapy; PC, pelvis cancer; VMAT, volumetric‐modulated arc therapy.

### 3.5 Analysis of %GP correlation between SunCHECK and Delta4

The %GP results for the same cases were obtained using PSQA in Delta4, and a correlation analysis was conducted between the Delta4 and SunCHECK results. The results are summarized in Table 10. In the HNC groups, there was a strong and highly significant correlation between the two sets of results (*r*: 0.73 ∼ 0.9, *p* < 0.01). Correlations in the TC groups were also very strong and significant (*r*: 0.73 ∼ 0.9, *p* < 0.01). However, in the PC groups, slight differences were observed between VMAT and IMRT. Specifically, at the dose standards of 3%/3  and 3%/2 mm, the correlations remained strong and highly significant; however, under the 2%/2  and 1%/1 mm criteria, the correlations were weaker, although still statistically significant.

**TABLE 10:** %GP correlation between SunCHECK and Delta4.

		3%3 mm		3%2 mm		2%2 mm		1%1 mm	
		VMAT	IMRT	VMAT	IMRT	VMAT	IMRT	VMAT	IMRT
HNC	*r*	0.9	0.73	0.87	0.78	0.85	0.82	0.73	0.86
	*p*	<0.01	<0.01	<0.01	<0.01	<0.01	<0.01	<0.01	<0.01
TC	*r*	0.9	0.73	0.87	0.78	0.85	0.82	0.73	0.86
	*p*	<0.01	<0.01	<0.01	<0.01	<0.01	<0.01	<0.01	<0.01
PC	*r*	0.75	0.89	0.55	0.83	0.6	0.76	0.57	0.8
	*p*	<0.01	<0.01	<0.01	<0.01	<0.01	<0.01	<0.01	<0.01

Abbreviations: %GP, gamma passing rate; HNC, head and neck cancer; IMRT, intensity‐modulated radiation therapy; PC, pelvis cancer; TC, thoracic cancer; VMAT, volumetric‐modulated arc therapy.

## 4 DISCUSSION

In this study, we aimed to assess the PSQA workflows of SunCHECK by following the guidelines outlined in the TG‐218 report. Additionally, we explored the establishment of personalized AL and TL for patients with cancers in varying anatomical regions. Further, we examined the relationship between SunCHECK's %GP and the clinical indicators of patients. Finally, the 3D %GPs obtained using SunCHECK were compared with Delta4 data to verify the reliability of the correlation analysis.

Analysis of the 3D %GP for 125 cases with various anatomical sites and treatment techniques determined the TLs and ALs based on the 3%/2 mm criteria. The results showed that using the 3%/2 mm standard from the TG‐218 report, the TLs of the HNC‐VMAT, TC‐VMAT, and PC‐VMAT groups were 94.65, 91.34, and 98.46, respectively, and the ALs were 93.68, 90.62, and 98.16. The TLs of the HNC‐IMRT, TC‐IMRT, and PC‐IMRT groups were 97.07, 95.72, and 97.31, and the ALs were 95.71, 94.80, and 96.79, respectively. Most of the TLs were higher than the 95% universal standard recommended by TG‐218. All ALs were higher than the 90% standard recommended by TG‐218. In addition, for different anatomical regions, not only were the TL and AL results different, but the VMAT and IMRT results also differed. The results indicated variability in TL and AL across different anatomical sites and between VMAT and IMRT techniques. Lower TL and AL were observed for thoracic tumors, suggesting that the plan complexity and the technological disparities between VMAT and IMRT may contribute to this variation.^17^, ^18^, ^22^ It is recommended to establish specific limits based on the anatomical site and radiation technique employed. The number of cases analyzed also had an important impact on the results; Fusella et al. demonstrated that AL and TL need to be regularly adjusted as the number of cases increases and new technologies are introduced.^23^

We conducted an analysis of %GP, which facilitated rapid evaluation of PSQA results. However, numerous studies also recommend considering additional clinically relevant factors, such as the correlation between the patient planning structure and the point of gamma analysis failure.^24^, ^25^ When the %GP failed to meet the TL and AL standards, a preliminary assessment was conducted to determine its clinical significance, considering whether there were any exceedances in the target area or vital organs.^11^ SunCHECK was utilized for calculations based on the patient's CT scan, enabling us to obtain related parameters and dose distributions from the DVH. The PTV %DE values of the HNC and TC groups were found to be higher than those of the other groups, potentially owing to the increased planning complexity and dose gradient observed in cases of nasopharyngeal carcinoma and multiple lung cancers, as well as the relatively lower planning complexity associated with cervical and rectal cancers.^26^, ^27^, ^28^, ^29^ Previous research has also indicated that when analyzed using 3DVH software, nasopharyngeal carcinoma cases exhibit a higher %DE than other cases.^30^ Among the OARs, the %DE in the lungs were the largest in the thoracic region plans because of the difference between the SDC of SunCHECK and AAA of TPS in the cavity calculation.^17^, ^18^ Simultaneously, the %DE of the PTV was strongly correlated with %GP, and the correlation was significant.

Therefore, the evaluation of %GP reflects some of the %DE results. However, when the PSQA does not meet the standard, the DVH and %DE should be checked. The TG‐218 report also mentioned that DVH analysis can be used to evaluate the clinical relevance of PSQA, especially when the %GP does not reach the TL. At the same time, Stasinou has shown that a patient quality control workflow should be established in the institution, including the %DE check of DVH parameters.^30^

Because SunCHECK performs PSQA based on accelerator log files and patient CTs, we synchronously evaluated its reliability and stability using Delta4 actual measurements.^31^, ^32^ This evaluation revealed a statistically significant and robust correlation, particularly at dose limits of 3%/3  and 3%/2 mm. Therefore, PSQA practices can be improved by incorporating actual measurements and log‐based evaluations. Our study encompassed 125 cases, which presents a limitation regarding the generalizability of our findings. The variations in accelerators and verification equipment used across different institutions could affect the universal applicability of our results. Conducting comprehensive studies across different institutions while considering variations in equipment and methods are recommended to enhance the generalizability of future findings.

## 5 CONCLUSION

In this study, the specific TL and AL limits of the VMAT and IMRT plans for the head, chest, and pelvis were calculated based on the workflow recommended in the TG‐218 report. The results showed that TL and AL were more stringent than the 95% and 90% standards of the common criterion of 3%/2 mm, respectively. In addition, clinical correlation analyses were performed between %GP and DVH‐based %DE, which revealed a stronger correlation for planning target volume structures and a weaker correlation for OARs structures. We also verified the reliability of the log file‐based software SunCHECK. In conclusion, we obtained TL and AL values for different patient sites and different treatment techniques at our center and recommend the use of a combination of clinically relevant metrics and %GP to assess PSQA outcomes.

## AUTHOR CONTRIBUTIONS

**Jia Deng** (First Author; Corresponding Author): Conceptualization; methodology; software; investigation; formal analysis; writing—original draft; funding acquisition; project administration. **Shengyan Liu**: Visualization; writing—original draft; validation. **Yun Huang**: Conceptualization; data curation; formal analysis; writing—original draft; writing‐review & editing. **Xiuquan Li**: Investigation; data curation; formal analysis; writing—original draft. **Xiangyang Wu**: Data curation; supervision; visualization; writing—review & editing.

## CONFLICT OF INTEREST STATEMENT

The authors have no conflicts to disclose.

## Supporting information

Supplementary information
