# Supplementary material for: Evaluating AAPM‐TG‐218 recommendations: Gamma index tolerance and action limits in IMRT and VMAT quality assurance using SunCHECK
Source: J Appl Clin Med Phys. 2024 Jan 19;25(6):e14277. doi: 10.1002/acm2.14277 (PMC11163510; doi:10.1002/acm2.14277)
Supplement: Supplementary file 1 — Supplementary information [file ACM2-25-e14277-s001.docx]

**Appendix 1**

**SunCHECK Universal Metrics QA Template**

|  | | Custom(1%/1mm) | Best(2%/2mm) | Better(3%/2mm) | Good(3%/3mm) |
| --- | --- | --- | --- | --- | --- |
| Diff(%) | | 1 | 2 | 3 | 3 |
| Dist(mm) | | 1 | 2 | 2 | 3 |
| Threshold(%) | | 10 | 10 | 10 | 10 |
| Passing(%) | | 95 | 95 | 95 | 95 |
| Nomalization | | Global | Global | Global | Global |
| 3D Analysis | **Targets** | Mean, D90%, D95% | | | |
|  | **OARs** | Mean, Max | | | |
